# *MPLW515L* Is a Novel Somatic Activating Mutation in Myelofibrosis with Myeloid Metaplasia

**DOI:** 10.1371/journal.pmed.0030270

**Published:** 2006-07-18

**Authors:** Yana Pikman, Benjamin H Lee, Thomas Mercher, Elizabeth McDowell, Benjamin L Ebert, Maricel Gozo, Adam Cuker, Gerlinde Wernig, Sandra Moore, Ilene Galinsky, Daniel J DeAngelo, Jennifer J Clark, Stephanie J Lee, Todd R Golub, Martha Wadleigh, D. Gary Gilliland, Ross L Levine

**Affiliations:** 1Brigham and Women's Hospital, Harvard Medical School, Boston, Massachusetts, United States of America; **2**Dana-Farber Cancer Institute, Harvard Medical School, Boston, Massachusetts, United States of America; **3**Broad Institute of Harvard and MIT, Cambridge, Massachusetts, United States of America; **4**Howard Hughes Medical Institute, Boston, Massachusetts, United States of America; University of California Los AngelesUnited States of America

## Abstract

**Background:**

The *JAK2V617F* allele has recently been identified in patients with polycythemia vera (PV), essential thrombocytosis (ET), and myelofibrosis with myeloid metaplasia (MF). Subsequent analysis has shown that constitutive activation of the JAK-STAT signal transduction pathway is an important pathogenetic event in these patients, and that enzymatic inhibition of JAK2V617F may be of therapeutic benefit in this context. However, a significant proportion of patients with ET or MF are *JAK2V617F-*negative. We hypothesized that activation of the JAK-STAT pathway might also occur as a consequence of activating mutations in certain hematopoietic-specific cytokine receptors, including the erythropoietin receptor (EPOR), the thrombopoietin receptor (MPL), or the granulocyte-colony stimulating factor receptor (GCSFR).

**Methods and Findings:**

DNA sequence analysis of the exons encoding the transmembrane and juxtamembrane domains of EPOR, MPL, and GCSFR, and comparison with germline DNA derived from buccal swabs, identified a somatic activating mutation in the transmembrane domain of MPL (W515L) in 9% (4/45) of *JAKV617F-*negative MF. Expression of MPLW515L in 32D, UT7, or Ba/F3 cells conferred cytokine-independent growth and thrombopoietin hypersensitivity, and resulted in constitutive phosphorylation of JAK2, STAT3, STAT5, AKT, and ERK. Furthermore, a small molecule JAK kinase inhibitor inhibited MPLW515L-mediated proliferation and JAK-STAT signaling in vitro. In a murine bone marrow transplant assay, expression of MPLW515L, but not wild-type MPL, resulted in a fully penetrant myeloproliferative disorder characterized by marked thrombocytosis (Plt count 1.9–4.0 × 10 ^12^/L), marked splenomegaly due to extramedullary hematopoiesis, and increased reticulin fibrosis.

**Conclusions:**

Activation of JAK-STAT signaling via MPLW515L is an important pathogenetic event in patients with *JAK2V617F-*negative MF. The bone marrow transplant model of MPLW515L-mediated myeloproliferative disorders (MPD) exhibits certain features of human MF, including extramedullary hematopoiesis, splenomegaly, and megakaryocytic proliferation. Further analysis of positive and negative regulators of the JAK-STAT pathway is warranted in *JAK2V617F-*negative MPD.

## Editors' Summary

**Background.:**

Myelofibrosis with myeloid metaplasia (MF) is one of a group of chronic blood disorders, known as chronic myeloproliferative disorders. These disorders sometimes turn into acute leukemia. The main abnormality in myelofibrosis is for the bone marrow to become filled with fibrous (scar) tissue (hence the name myelofibrosis), which stops it from producing normal blood cells efficiently. In addition, the white blood cells that remain are abnormal (that is, metaplastic). The clinical effect of these abnormalities are that patients are anemic (they have low numbers of red cells), are more likely to get infections because of the abnormal white cells which cannot fight infections normally, and may bleed more easily because of a lack of the platelets that help the blood to clot. Scientists who study this disorder believe that the disease starts from just one abnormal cell, which divides to replace all the other cells—that is, all the abnormal cells are part of one clone.

**Why Was This Study Done?:**

In two similar diseases, polycythemia vera (in which the bone marrow produces too many red blood cells) and essential thrombocytosis (in which the bone marrow produces too many platelets), and in some patients with MF, scientists have found genetic changes which seem to trigger these diseases. However, there are some patients with MF in which no abnormal gene has been found. The scientists here wanted to look at other genes to see if they could find any changes that might trigger MF.

**What Did the Researchers Do and Find?:**

They decoded the DNA sequence of three genes that are known to be involved in how blood cells develop for 45 patients with MF. They looked at DNA from white blood cells, and also from normal cheek cells for comparison. They found that in four of the 45 patients the DNA in the bone marrow, but not the cheek, carried a mutation in a gene for the thrombopoietin receptor (also called *MPL*). This gene is necessary for the cells that make platelets to grow correctly. The mutation was not present in any samples from patients with diseases related to MF, nor in 270 normal samples. The mutation that was identified was at position 515 in the *MPL* gene sequence, hence the name MPLW515L—the W and the L are the shorthand way of indicating exactly which change occurred. The change meant that the gene became abnormally active. The researchers tested the effect of the abnormal gene by putting it into cells grown in culture in the laboratory; they found that it made the cells grow more than was normal. In addition, when cells with the abnormal gene were put into mice, the mice developed a blood disorder similar to that seen in humans with MF.

**What Do These Findings Mean?:**

It seems likely that the genetic change that has been identified here is responsible for the MF that develops in some patients. The *MPL* gene is known to be part of a pathway of genes that control how certain blood cells develop. However, it is not yet clear exactly how the genetic change found here causes the blood cells to grow abnormally, or how it causes the other clinical effects of MF. Further work will also need to be done to see if it is possible to develop drugs that can act on this gene mutation, or on the other genes that it affects so as to return the cells to normal.

**Additional Information.:**

Please access these Web sites via the online version of this summary at http://dx.doi.org/10.1371/journal.pmed.0030270.

• MedlinePlus, a Web site of the US National Library of Health, has pages of information on myelofibrosis and related diseases

• The National Cancer Institute, which funds research into many cancers, has information for patients on myelofibrosis, including information on clinical trials

• The MPD Foundation has information for patients with myelofibrosis and related diseases

## Abstract 3

Activation of JAK-STAT signaling via a mutation - MPLW515L- in the thrombopoietin receptor seems to have a role in the pathogenesis of some patients with myelofibrosis.

## Introduction

The BCR-ABL negative chronic myeloproliferative disorders (MPD) include polycythemia vera (PV), essential thrombocytosis (ET), and myelofibrosis with myeloid metaplasia (MF) [ 1]. Although clonal hematopoiesis was observed in these disorders more than three decades ago, the molecular etiology of these disorders was not known until recently when several groups reported a somatic mutation in the JAK2 tyrosine kinase ( *JAK2V617F*) in most patients with PV and in a subset of patients with ET and MF [ 2– 6]. Recent estimates using high sensitivity mutation detection techniques indicate that *JAK2V617F* is present in ˜95–100% of PV, 60%–70% of ET, and 50% of MF [ 7, 8]. JAK2V617F is a constitutively active tyrosine kinase [ 9] that activates downstream signal transduction pathways and transforms hematopoietic cells to cytokine-independent growth [ 4, 10], and these cells are sensitive to a small molecule JAK Inhibitor [ 2]. In addition, expression of JAK2V617F in a murine bone marrow transplant assay results in a MPD most similar to PV [ 4, 11]. These data indicate that constitutive activation of JAK-STAT signaling by the mutant JAK2V617F kinase plays a central role in the pathogenesis of *JAK2V617F-*positive PV, ET, and MF.

Despite the recent discovery of the *JAK2V617F* allele, questions remain regarding the molecular pathogenesis of PV, ET, and MF. In particular, the mutation(s) responsible for *JAK2V617F-*negative ET and MF remain to be identified. Although more sensitive techniques can identify *JAK2V617F* mutations in a small proportion of clonal cells, we recently demonstrated that the majority of *JAK2V617F-*negative ET and MF patients have clonal granulocytes [ 7], suggesting that alternative mutation(s) result in clonal granulopoiesis in this subset of patients.

We hypothesized that *JAK2V617F-*negative patients with MPD might have mutations involving other components of the JAK-STAT signal transduction pathway that include cytokine receptors, other JAK family members, or STAT family members. Screens for additional mutations in other JAK-STAT signaling intermediates in this clinical context including *JAK1*, *JAK3*, *TYK2*, *STAT3*, or *STAT5* have been negative [ 12].

We have recently shown that expression of a homodimeric type I cytokine receptor, such as the erythropoietin receptor (EPOR), the thrombopoietin receptor (MPL), or the granulocyte-colony stimulating factor receptor (GCSFR), is required for JAK2V617F-mediated transformation of hematopoietic cells and for activation of downstream signaling [ 10]. These data suggested the possibility that mutations in the regions of these cytokine receptors that are critical for receptor dimerization (transmembrane domain) and for JAK2 binding (juxtamembrane domain) might lead to activation of JAK-STAT signaling in *JAK2V617F-*negative MPD. Indeed, such mutations in the context of *EPOR* have been identified in rare familial cases of polycythemia, though these have not been reported in acquired MPD. Heretofore, *MPL* has been sequenced in a small cohort of patients with MF and ET, but no mutations were identified [ 13], and multiple groups have reported the absence of *EPOR* mutations in small numbers of patients with PV [ 14, 15]. High throughput DNA sequence analysis and the collection of a large number of MPD patient samples [ 2] has enabled evaluation of a larger series of patients for mutations in candidate genes, including cytokine receptors. We therefore investigated patients with *JAK2V617F-*negative MPD for somatic activating mutations in *EPOR, MPL,* or *GCSFR*.

## Methods

### Sample Collection

Granulocyte DNA samples and matched normal DNA samples were collected from patients with PV, ET, and MF who were enrolled in the Harvard Myeloproliferative Disorders Study as previously described [ 2]. Additional samples from patients with MF were collected on a separate Dana-Farber Cancer Institute protocol. All subjects provided informed consent on protocols approved by the Dana-Farber Cancer Institute Institutional Review Board.

### DNA Sequence Analysis and Genotyping for *MPLW515L*

PCR amplification and DNA sequencing of select exons of *MPL, EPOR,* and *GCSFR* was performed using M13-tailed primers as previously described [ 2], and specific primer sequences are listed in Table S1. Sequence analysis of bidirectional sequence traces was performed using Mutation Surveyor version 2.28 (SoftGenetics, State College, Pennsylvania, United States). Candidate mutations were reamplified and sequenced from original DNA for independent verification, and sequence analysis of buccal DNA was performed to ascertain whether non-synonymous mutations were constitutional or somatic in origin. Identity between granulocyte and buccal DNA for individual patients was confirmed using eight informative synonymous single nucleotide polymorphisms. Genotypic analysis of the HapMap panel of normal patients was performed using a mass spectrometric assay as previously described [ 2].

### Expression Vectors and Cell Culture

The MSCV-MPL-Neo and MSCV-MPL-IRES-EGFP retroviral vectors were generously provided by W. Tong and H. Lodish. The *MPLW515L* mutation was generated using site-directed mutagenesis (Quickchange-XL, Stratagene, La Jolla, California, United States) and confirmed by full-length DNA sequencing. 293T cells were grown in DMEM with 10% fetal bovine serum (FBS). Transient co-transfection of 293T cells and generation of retroviral supernatant were performed using Fugene (Roche, Nutley, New Jersey, United States) according to manufacturer's guidelines. 32D and Ba/F3 cells were grown in RPMI medium 1640 containing 10% FBS and 10% WEHI-3B cell supernatant as a source of IL3. UT7 cells were grown in IMDM medium supplemented with 10% FBS and GMCSF (1 ng/mL). 32D cells, Ba/F3 cells, and murine bone marrow cells were transduced with viral supernatant, and UT7 cells were transduced by electroporation (Amaxa, Gaithersburg, Maryland, United States). Cells were then selected in G418 (1mg/mL). To assess for factor-independent growth and for thrombopoietin hypersensitivity, cells were first washed three times in PBS, and then 1 × 10 ^5^ viable cells/mL were resuspended in appropriate cytokine-free media or in media containing different concentrations of thrombopoietin (Sigma, St. Louis, Missouri, United States) as noted. The number of viable cells was determined by trypan blue exclusion.

### Western Blot Analysis

Cells were collected and lysed in lysis buffer and separated by electrophoresis as described previously [ 16]. Nitrocellulose membrane was blocked in TBST/5% milk and incubated with one of the following antibodies: anti-pSTAT5 (Cell Signaling, Beverly, Massachusetts, United States), anti-pSTAT3 (Cell Signaling), anti-pERK (Cell Signaling), anti-pAKT (Cell Signaling), anti-pTYK2 (Cell Signaling), or anti-pJAK2 (Cell Signaling). For loading controls, blots were stripped and reprobed using anti-JAK2 (Santa Cruz Biotechnology, Santa Cruz, California, United States), anti-TYK2 (Santa Cruz Biotechnology), anti-STAT5 (Santa Cruz Biotechnology), anti-STAT3 (Cell Signaling), anti-ERK (Cell Signaling), anti-AKT (Cell Signaling), and anti-TYK2 (Santa Cruz Biotechnology) antibodies as appropriate.

### JAK Inhibitor Assays

For cell-proliferation assays, 32D cells stably expressing FIP1L1-PDGFRA [ 16] and MPLW515L were grown in RPMI/10% FBS and then incubated with appropriate concentrations of JAK Inhibitor I (Calbiochem, San Diego, California, United States) for 72 hours. 32D cells expressing MPLWT were grown in RPMI/10%FBS/mTPO in the presence of JAK Inhibitor I. The number of viable cells was determined using CellTiter 96 Aqueous One Cell Proliferation Assay (Promega, San Luis Obispo, California, United States). For Western blotting, cell lines were incubated in the presence of JAK Inhibitor I for four hours, and phosphorylation of relevant proteins was assessed as described above.

### Murine Bone Marrow Transplant Assay

The murine bone marrow transplant assay was performed as previously described [ 17]. MSCV retroviral supernatants were titered by determining the percentage of GFP positive cells 48 hours after infection of Ba/F3 and 32D cells (1 mL supernatant used to infect 1 × 10 ^6^ cells). MPLWT and MPLW515L retroviral supernatants were able to reproducibly infect 40%–60% of Ba/F3 and 32D cells as assessed by flow cytometry. Balb/C donor mice were treated with 5-Florouracil (150 mg/kg) seven days prior to bone marrow harvest. Bone marrow cells were harvested from donor mice, treated with red blood cell lysis buffer and cultured for 24 hours in transplantation medium (RPMI + 10% FBS + 6 ng/mL IL-3, 10 ng/mL IL-6, and 10 ng/mL stem-cell factor (SCF)). Cells were treated by spin infection with retroviral supernatants (1 mL supernatant per 4 × 10 ^6^ cells, plus polybrene) and centrifuged at 1,800 *g* for 90 minutes. The spin infection was repeated 24 hours later. The cells were then washed and resuspended in Hank's balanced salt solution, and injected into lateral tail veins of lethally irradiated (2 × 4.5 Gy [450 rad]) Balb/C recipient mice (Taconic, Germantown, New York, United States) at 0.5 to 1.0 × 10 ^6^ cells/mouse. Animals were humanely killed when they had palpable splenomegaly or were moribund. Animals transplanted with bone marrow that was transduced with MPLWT viral supernatant were killed at the time of manifestation of MPLW515L disease for endpoint analysis.

### Mouse Analysis

Peripheral blood was collected from the retro-orbital cavity using EDTA-treated glass capillary tubes and analyzed by automated complete and differential blood cell counts and blood smears (Wright-Giemsa stained). Single-cell suspensions of spleen and bone marrow were prepared by pressing tissue through a cell strainer, followed by red blood cell lysis. For histopathology, tissues were fixed in formalin and then embedded in paraffin for histology analysis as previously described [ 18].

For flow cytometry, cells were washed in PBS + 1% bovine serum albumin and stained with monoclonal antibodies in PBS + 1% bovine serum albumin for 30 minutes on ice. Antibodies used were allophycocyanin–conjugated Gr-1, B220, Ter119, CD8, cKit; and phycoerythrin–conjugated Mac-1, CD19, CD71, CD4, CD41 (BD Pharmingen, San Diego, California, United States). Flow cytometry analysis was performed on a FACSCalibur instrument (BD Biosciences, San Jose, California, United States) and analyzed with CellQuest software (BD Biosciences). Viability was assessed by incubating cells with 7-AAD (7-amino-actinomycin D) (BD Pharmingen) for five minutes prior to flow cytometry. Cells were gated for viability (using forward/side scatter and 7-AAD) and GFP positivity, and 10,000 events were analyzed from this subset for marker expression.

The study protocol was reviewed and approved by the Boston (Massachusetts, United States) Children's Hospital Animal Care and Use Committee.

### In Vitro Colony-Forming Assays

Myeloid colony–forming assays were performed in methylcellulose-based medium (M3434) containing 3U/mL erythropoietin, 10 ng/mL IL-3, 10 ng/mL IL-6, and 50 ng/mL SCF, and in M3231 medium containing no additional cytokines, as per manufacturer's protocols (StemCell Technologies, Vancouver, British Columbia, Canada). Cells were plated at 1.5 × 10 ^4^ cells per dish for bone marrow cells and 5 × 10 ^4^ cells per dish for spleen cells, in duplicate. Plates were incubated at 37 °C for seven days. Colonies were scored by morphology, and representative colonies were confirmed by Wright-Giemsa stained slides of colony cytospins. Graphs represent total number of colonies counted for three representative mice in each group, with two plates counted for each mouse. Megakaryocyte colony–forming assays were performed in collagen-containing medium (MegaCult-C; StemCell Technologies) containing TPO, IL-11, IL-3, and IL-6 for seven days. Slides were fixed and stained for six hours with acetylcholine iodide (Sigma) as per MegaCult protocol, and were counterstained with Harris hematoxylin solution (Sigma) as per manufacturer's protocol.

To assess megakaryocyte ploidy, bone marrow cells were cultured for four days in RPMI/10% FBS containing 50 ng/mL mTPO and 10 mg/mL SCF. Cells were collected and stained with FITC-rat anti-mouse CD41 antibody (BD Biosciences), followed by 50 ug/mL propidium iodide in 0.1% sodium citrate buffer with 50 ug/mL RNAase, as previously described [ 19]. Data was acquired on a FACSCalibur (BD Biosciences) and analyzed with CellQuest software.

## Results

### *MPLW515L* Mutation in JAK2 *V617F*-Negative MPD

**Figure 1:**
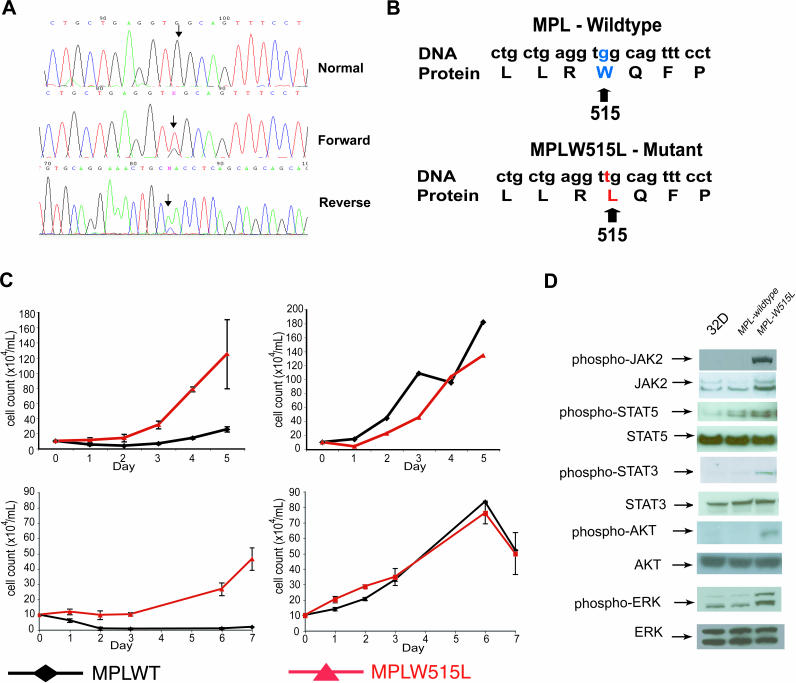
MPLW515L Mutation Is Found in JAK2V617F-Negative MF and Causes Cytokine-Independent Growth in 32D and UT7 Cells, and Constitutively Activates the JAK-STAT Signaling Pathway (A) Forward (middle trace) and reverse (lower trace) sequence traces demonstrating a heterozygous guanine to thymine substitution (arrows) present in granulocyte DNA from a patient with MF. The mutation is not present in buccal DNA from the same patient (upper trace). (B) DNA sequence and protein translation for both the wild-type and mutant MPL alleles. The mutation results in a tryptophan-to-leucine substitution at codon 515. (C) *Upper:* 32D cells transduced with MPLW515L exhibit cytokine-independent growth compared with MPLWT (left). Cell lines grown in the presence of IL3 show equal rates of growth (right). Error bars denote the standard deviation for each sample measured in triplicate. *Lower:* UT7 cells transformed with MPLW515L exhibit cytokine-independent growth compared with MPLWT (left). Cell lines grown in the presence of TPO (5 ng/mL) show equal rates of growth (right). Error bars denote the standard deviation for each sample measured in triplicate. (D) 32D cells, 32D MPLWT cells, and 32D MPLW515L cells were deprived of cytokines and then analyzed by Western blots, demonstrating phosphorylation of JAK2, STAT5, STAT3, AKT, and ERK in MPLW515L compared with MPLWT.

We sequenced the transmembrane and juxtamembrane domains of *EPOR, GCSFR,* and *MPL* in 15 *JAK2V617F*-negative MF patients, 16 *JAK2V617F*-negative patients with ET, and 4 *JAK2V617F*-negative PV patients. Although we found no mutations in *EPOR* or *GCSFR,* we identified a guanine to thymidine substitution in *MPL* at nucleotide 1544 which results in a tryptophan to leucine substitution at codon 515 ( *MPLW515L*) in two patients with MF ( Figure 1A and 1B). Sequencing of 30 additional *JAK2V617F-*negative MF samples identified two additional *MPLW515L*-positive samples, for a total frequency of 4/45 (9%) patients. *MPLW515L* was not detected in any JAK2 *V617F*-negative ET ( *n* = 50) or PV ( *n* = 10) patient samples. Sequence analysis of matched normal DNA derived from buccal swabs did not identify the *MPLW515L* substitution ( Figure 1A, upper trace), demonstrating that *MPLW515L* is a somatic mutation in hematopoietic cells. Sequence analysis of the entire open reading frame of *MPL* in all *JAK2V617F-*negative ET and MF patients did not reveal additional somatic mutations. To assess the prevalence of the *MPLW515L* allele in the general population, we genotyped a standard panel of 270 samples collected by the International HapMap Consortium [ 20]. All 270 HapMap samples were homozygous for the wild-type *MPLW515* allele.

No significant clinical differences were observed between *MPLW515L*-positive versus *MPLW515L*-negative patients with MF with regard to median age at presentation and to disease duration, though the statistical power of this analysis was limited by the small number of patients with MF in our cohort. For all *MPLW515L-*positive MF patients for which bone marrow histopathology was available (3/4), marked megakaryocyte hyperplasia was noted. In addition, two of the four *MPLW515L-*positive patients exhibited leukocytosis and thrombocytosis at the time of disease presentation. One of the four *MPLW515L-*positive patients had a rapidly progressive clinical course, with death two years after initial presentation from complications related to MF.

### Functional Analysis of MPLW515L and Wild-Type MPL

**Figure 2:**
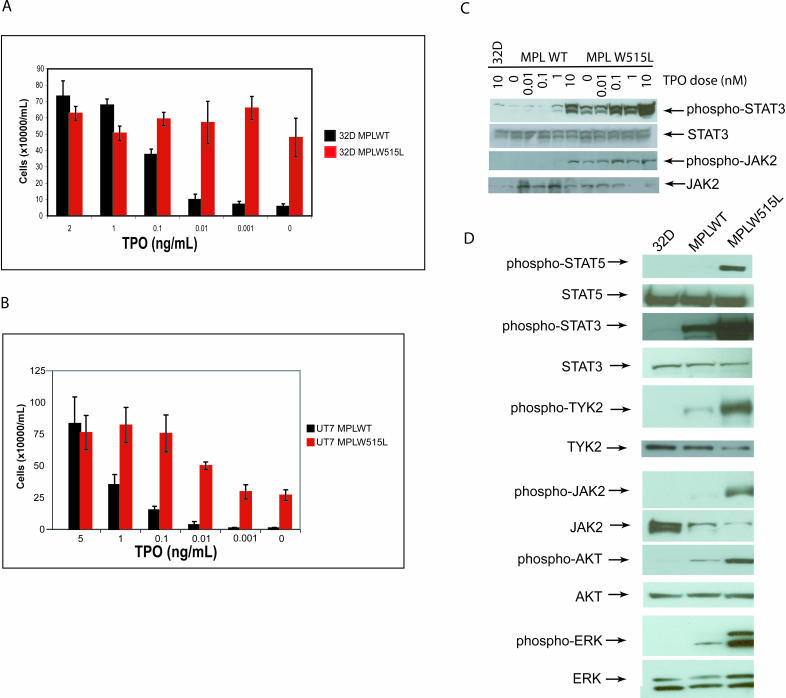
MPLW515L-Expressing Cells Show Hyper-Responsiveness to TPO (A) 32D MPLWT cells and 32D MPLW515L cells were cultured in triplicate at an initial concentration of 1 × 10 ^5^ cells/mL, in RPMI/10% FBS containing TPO 2 ng/mL, 1 ng/mL, 0.1 ng/mL, 0.01 ng/mL, 0.001 ng/mL, or in the absence of TPO for four days, and then cell numbers were assessed. Error bars denote the standard deviation for each sample measured in triplicate. (B) UT7 MPLWT cells and UT7 MPLW515L cells were cultured in triplicate, at an initial concentration of 1 × 10 ^5^ cells/mL, in IMDM/10% FBS containing TPO 5 ng/mL, 1 ng/mL, 0.1 ng/mL, 0.01 ng/mL, 0.001ng/mL, or in the absence of TPO for six days, and then cell numbers were assessed. Error bars denote the standard deviation for each sample measured in triplicate. (C) 32D MPLWT or 32D MPLW515L cells were deprived of cytokine overnight and then stimulated with TPO 5 ng/mL, 1 ng/mL, 0.1 ng/mL, 0.01 ng/mL, or without TPO for seven minutes and then analyzed by Western blot for phosphorylation of JAK2 and STAT3, demonstrating increased JAK2/STAT3 phosphorylation in response to TPO in 32D MPLW515L cells as compared with 32D-MPLWT cells. (D) Phosphorylation of JAK2, TYK2, STAT3, STAT5, AKT, and ERK in response to seven-minute stimulation with TPO (50ng/mL). 32D MPLWT or 32 MPLW515L cells were deprived of cytokine overnight and then stimulated with TPO 50 ng/mL for seven minutes and then analyzed by Western blot for phosphorylation of JAK2, TYK2, STAT3, STAT5, AKT, and ERK, demonstrating increased phosphorylation of these proteins in response to TPO in 32D MPLW515L cells as compared with 32D MPLWT cells.

In cell culture assays, expression of MPLW515L, but not MPLWT, conferred cytokine-independent growth to 32D, UT7, or Ba/F3 cell lines ( Figure 1C and unpublished data). Western blotting demonstrated constitutive phosphorylation of the JAK/STAT signaling proteins, including JAK2, STAT3, and STAT5 ( Figure 1D). Downstream targets of this pathway were also activated, with constitutive phosphorylation of p42/44 ERK and AKT in 32D MPLW515L cells consistent with activation of the RAS/MAPK and PI3K/AKT pathways ( Figure 1D). In addition, expression of MPLW515L in 32D and UT7 cells resulted in hypersensitivity to TPO compared with MPLWT as assessed by cell growth ( Figure 2A and 2B) and by Western blotting after TPO stimulation ( Figure 2C and 2D).

### A Murine Model for MPLW515L-Induced Myeloproliferative Disease

**Figure 3:**
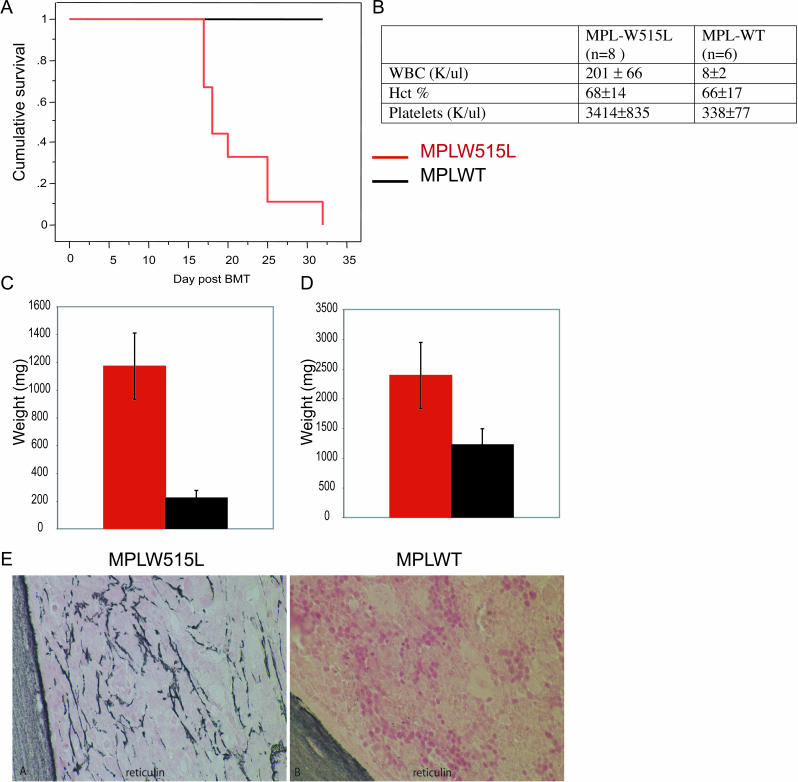
Bone Marrow Transplant with MPLW515L-Transduced Bone Marrow Causes a Rapid Myeloproliferative Disease (A) Kaplan-Meier survival plot of Balb/C mice transduced with MPLW515L ( *n* = 9) and MPLWT ( *n* = 6) showing death of all MPLW515L mice between 17 and 32 days post-BMT compared with MPLWT ( *n* = 6), which were sacrificed for endpoint analysis without evidence of disease. (B) Complete blood counts show leukocytosis and thrombocytosis in MPLW515L BMT model compared with MPLWT. There is no difference in hematocrits. Standard deviation is indicated. (C) Spleen weights of MPLW515L and MPLWT mice shows splenomegaly in MPLW515L mice but not in MPLWT mice with average spleen weight equal to 1,171 mg. (D) Liver weights of MPLW515L and MPLWT mice shows hepatomegaly in MPLW515L mice but not in MPLWT mice, with average liver weight equal to 2,390 mg (compared with 1,222 mg in MPLWT mice). (E) Bone marrow shows significantly increased bone marrow fibrosis by reticulin staining at 17 days post-BMT in MPLW515L mice, but not in MPLWT-expressing mice.

To assess the in vivo effects of the MPLW515L mutant receptor, we developed a murine model of MPLW515L-induced myeloproliferative disease. Bone marrow cells were transduced with MSCV-IRES-EGFP vectors containing MPLWT or MPLW515L and transplanted into lethally irradiated Balb/C mice. MPLW515L-transduced animals, but not MPLWT-transduced animals, developed a short latency (median latency = 18 days post BMT), fully penetrant, lethal MPD ( Figure 3A) notable for marked thrombocytosis (mean platelet count = 3.414 × 10 ^12^/L) as well as leukocytosis (mean WBC = 201 × 10 ^9^/L) ( Figure 3B). There was no significant difference in the hematocrit of MPLW515L mice compared with MPLWT mice. At the time of sacrifice, MPLW515L-expressing mice showed splenomegaly and hepatomegaly ( Figure 3C and 3D), and staining of bone marrow demonstrated increased reticulin fibrosis in MPLW515L, but not MPLWT-expressing mice ( Figure 3E).

**Figure 4:**
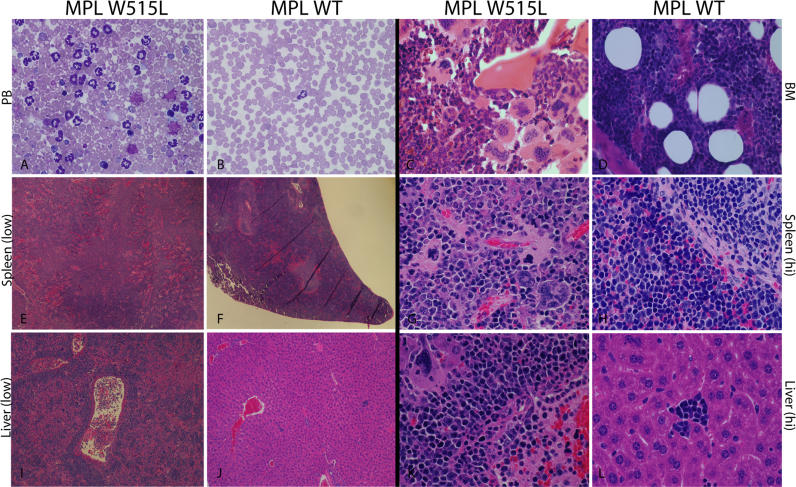
MPLW515L and MPLWT Bone Marrow Transplant Model Histopathology Histology of MPLW515L-transduced and MPLWT-transduced Balb/C mice showing images of peripheral blood (A and B) and histopathology in representative sections of bone marrow (C and D), spleen (E–H), and liver (I–L). Peripheral blood smear (B) (600×, Wright-Giemsa) of a representative MPLWT animal displays an unremarkable white blood cell and platelet count. In contrast, peripheral blood smear (A) (600×, Wright-Giemsa) of a representative MPLW515L mutant animal reveals marked thrombocytosis and leukocytosis comprising a predominant population of maturing myeloid cells as well as frequent nucleated erythroid forms. Bone marrow images from MPLWT animals display preserved marrow architecture with maturing trilineage hematopoiesis (D) (600×, hematoxylin and eosin [H&E]). Comparatively, bone marrow sections from MPLW515L mutant animals demonstrate marrow elements comprising a prominent population of maturing myeloid cells with increased numbers of megakaryocytes including atypical and dysplastic forms occurring in frequent clusters (C) (600×, H&E) and showing emperipolesis of neutrophils in megakaryocyte cytoplasm. Spleen sections from MPLW515L mice display complete effacement of normal splenic architecture (E) (40×, H&E) with a marked expansion of red pulp that is composed of an admixture of maturing myeloid and erythroid elements and numerous numbers of atypical megakaryocytes (G) (600×, H&E) compared with MPLWT spleens (F and H) (40× and 600×, H&E), which display a relative preservation of normal spleen architecture and the presence of only maturing erythroid forms in the red pulp. Liver images from MPLW515L mice illustrate evidence of extensive extramedullary hematopoiesis in a perivascular and sinusoidal distribution (I) (100×, H&E) composed predominantly of a population of maturing erythroid elements with frequent large atypical megakaryocytes and occasional admixed myeloid forms (K) (600×, H&E). In comparison, only small, focal areas of nucleated erythroid cells were observed in livers from MPLWT animals (J and L) (100× and 600×, H&E).

Histopathology analysis was consistent with the presence of a MPD in MPLW515L-expressing mice, but not in MPLWT-expressing mice ( Figure 4). Examination of peripheral blood smears from MPLW515L mice revealed marked thrombocytosis and leukocytosis composed of predominantly mature myeloid elements admixed with occasional to frequent numbers of nucleated erythroid cells ( Figure 4A and 4B). Compared with MPLWT animals, MPLW515L mice demonstrated markedly hypercellular bone marrow with a predominance of maturing myeloid forms and with increased numbers of atypical and dysplastic megakaryocytes which could be found in frequent clusters and exhibited emperipolesis of neutrophils in megakaryocyte cytoplasm ( Figure 4C and 4D). Spleens from MPLW515L mice revealed complete effacement of normal splenic architecture ( Figure 4E), with a prominent expansion of red pulp composed of an admixture of maturing myeloid and erythroid elements, and numerous abnormal megakaryocytes. By comparison, spleens from MPLWT animals displayed a relative preservation of normal splenic architecture with only a mild expansion of red pulp by maturing erythroid elements ( Figure 4F and 4H) that were also noted in small, focal clusters in the livers of these animals ( Figure 4J and 4L). In contrast, extensive extramedullary hematopoiesis was observed in the livers of MPLW515L animals, which was composed primarily of maturing erythroid elements with frequent atypical megakaryocytes and relatively fewer numbers of mature myeloid cells ( Figure 4I and 4K).

**Figure 5:**
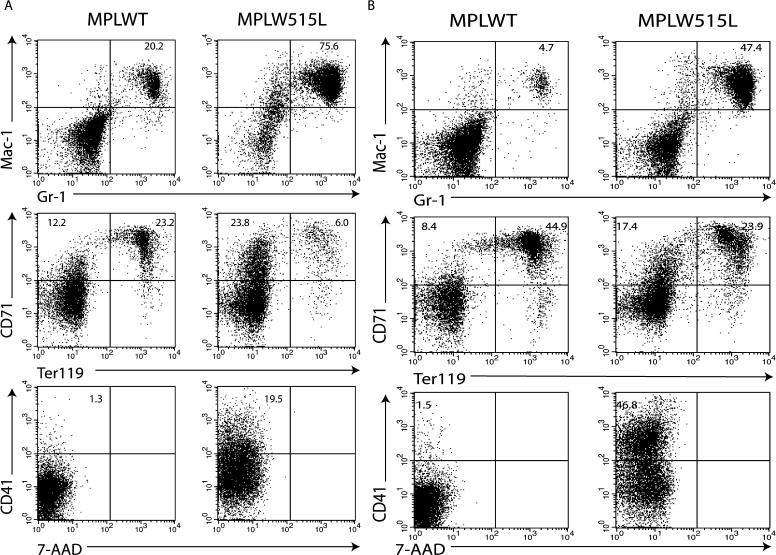
Flow Cytometry Analysis of BM and Spleen in Mice Transduced with MPLW515L and MPLWT (A) Flow-cytometry analysis of bone marrow cells shows a 4-fold increase in Mac1+/Gr1+ cells and a shift to a more immature erythroid population. There is a 15-fold increase in CD41+ cells in bone marrow expressing MPLW515L compared with MPLWT. (B) Flow-cytometry analysis of spleen cells shows a 10-fold increase in Mac1+/Gr1+ cells in MPLW515L. There is also a shift to a more immature erythroid population in MPLW515L with a greater percentage of CD71+/Ter119- cells. CD41+ cells are increased 30-fold in MPLW515L spleen cells compared with MPLWT spleen cells.

Consistent with the histopathologic findings, flow cytometry analysis of MPLW515L bone marrow cells demonstrated an ˜4-fold increase in Mac1+/Gr1+ mature myeloid cells as compared with bone marrow cells from MPLWT animals ( Figure 5A, upper panels). These findings were also reflected in the splenocyte populations examined from MPLW515L animals that revealed a nearly 10-fold increase in Mac1+/Gr1+ cells versus MPLWT spleens ( Figure 5B, upper panels). Further analysis of MPLWT and MPLW515L spleen and bone marrow cells demonstrated a significant erythroid population ( Figure 5B, middle panel) in both sets of animals, although a shift to a more immature erythroid population was observed in MPLW515L compared with MPLWT mice as reflected by an ˜2-fold increase in CD71+/Ter119- cells ( Figure 5A and 5B, middle panels). The marked increase in megakaryocytes seen in the histopathology of MPLW515L mice was corroborated by an ˜15-fold increase in the percentage of CD41+ cells in MPLW515L bone marrow compared with MPLWT ( Figure 5A, bottom panels). Similar increases were observed in MPLW515L spleen cells compared with MPLWT with an ˜30-fold increase in the proportion of CD41+ cells observed in MPLW515L spleen cells compared with MPLWT ( Figure 5B, bottom panels). Analysis of B cells demonstrated a proportionate decrease in B220 positive cells in MPLW515L bone marrow and spleen (unpublished data). There was no difference in CD4/CD8+ or c-Kit positive cell populations between MPLW515L and MPLWT bone marrow and spleen cells (unpublished data).

### MPLW515L Confers a Proliferative Advantage to Bone Marrow and Spleen Cells

**Figure 6:**
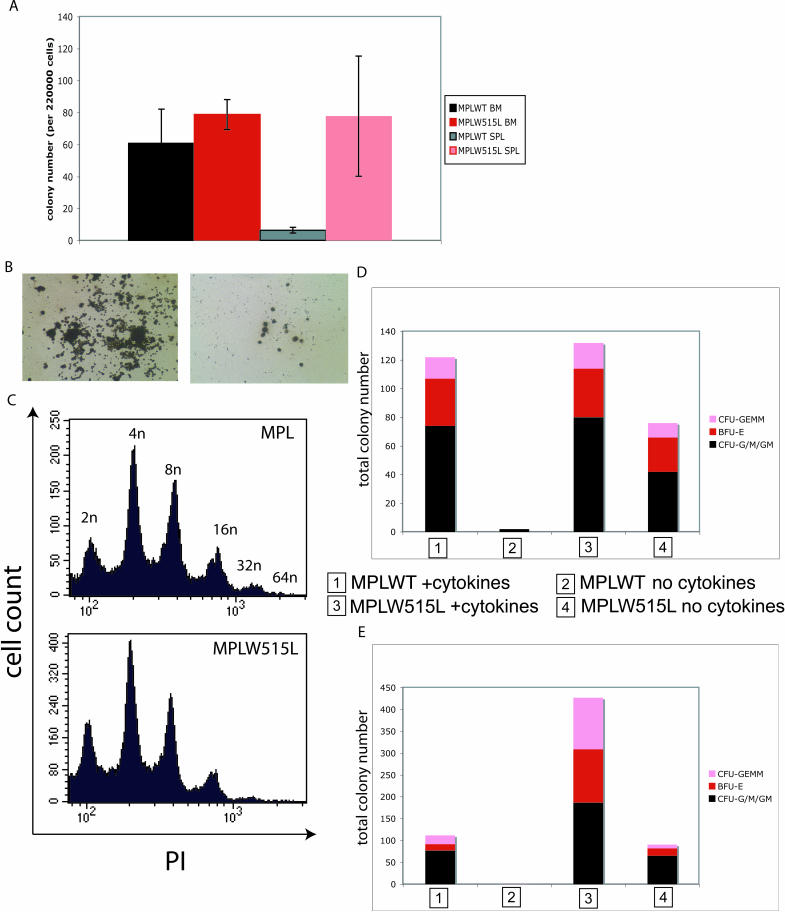
MPLW515L Increases the Number of Megakaryocyte and Myeloid Colonies in Spleen, without Affecting Megakaryocyte Ploidy, and Causes Cytokine-Independent Myeloid Colony Growth (A) Megakaryocyte colony–forming assay in the presence of TPO, IL3, IL11, and IL6 demonstrates similar numbers of megakaryocyte colonies obtained from MPLW515L-expressing bone marrow and an increase in the number of megakaryocyte colonies from spleen cells compared with MPLWT. (B) Acetylcholinesterase staining of megakaryocyte colonies derived from bone marrow demonstrates much larger colony size in MPLW515L as compared with MPLWT megakaryocyte colonies. (C) Megakaryocyte ploidy analysis shows the same distribution for MPLW515L-expressing cells and MPLWT-expressing cells. (D and E) Total myeloid colony formation from bone marrow cells (D) and spleen cells (E) demonstrates cytokine-independent colony formation in MPLW515L bone marrow and spleen. There is no difference in colony distribution between MPLWT- and MPLW515L-expressing cells. Colony counts reflect only positively identifiable colonies, with thorough megakaryocyte colony analysis done in MegaCult assay ( Figure 6A), and thus are excluded from methylcellulose colony analysis. Colony numbers represent a total of three representative mice per group, in duplicate.

Megakaryocyte colony–forming assays of bone marrow cells derived from MPLW515L- and MPLWT-expressing mice demonstrated a similar number of megakaryocyte colony-forming units, although there was a marked increase in megakaryocyte colony–forming units formed from spleen cells from MPLW515L-expressing mice compared with MPLWT ( Figure 6A). In addition, acetylcholinesterase staining demonstrated MPLW515L megakaryocyte colonies ( Figure 6B, left picture) that were significantly larger than MPLWT megakaryocyte colonies ( Figure 6B, right picture). The difference in megakaryocyte colony growth and morphology was not explained by a difference in ploidy of megakaryocyte cells in MPLW515L compared with MPLWT bone marrow cells ( Figure 6C). Perhaps the most striking finding was the ability of bone marrow or spleen cells derived from MPLW515L animals, but not MPLWT, to grow in methylcellulose culture in the absence of any cytokines ( Figure 6D and 6E). There was also an increase in the number of myeloid colonies in methylcellulose supplemented with cytokines from MPLW515L-expressing spleen cells ( Figure 6E), but not bone marrow cells ( Figure 6D), consistent with a shift in hematopoiesis from the bone marrow to the spleen in MPLW515L-expressing mice. There was no difference in the distribution of myeloid colonies grown from MPLWT- and MPLW515L-expressing mice ( Figure 6D and 6E).

### JAK Kinase Inhibition Results in Inhibition of MPLW515L-Induced Proliferation and Signal Transduction

**Figure 7:**
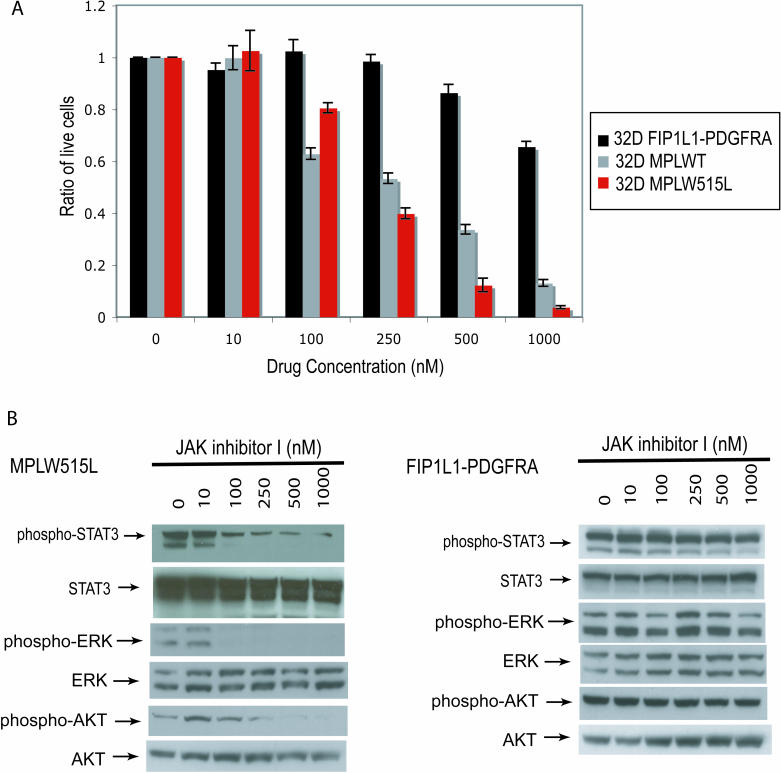
32D MPLW515L Cells Are Sensitive to JAK Inhibitor I (A) Dose-dependent inhibition of growth of 32D MPLW515L and 32D MPLWT cells but not 32D FIP1L1-PDGFRA cells with increasing doses of JAK Inhibitor I. (B) Reduction in STAT3 phosphorylation in 32D MPLW515L cells but not 32D FIP1L1-PDGFRA cells with increasing doses of JAK Inhibitor I. Cells were incubated with varying drug concentrations for four hours and then collected for Western blot analysis.

Treatment of 32D MPLW515L or 32D MPLWT cells but not 32D FIP1L1-PDGFRA cells with a small molecule JAK kinase inhibitor [ 21] resulted in a dose-dependent inhibition of cell growth ( Figure 7A). Dose-dependent inhibition of phosphorylation of STAT3, ERK, and AKT was observed in 32D MPLW515L and MPLWT cells but not in 32D FIP1L1-PDGFRA cells ( Figure 7B; unpublished data). These data indicate that MPLW515L-mediated proliferation and activation of JAK-STAT signaling is sensitive to inhibition with a small molecule JAK kinase inhibitor.

## Discussion

In this report we describe a somatic mutation in the transmembrane region of *MPL* ( *MPLW515L*) in a subset of *JAK2V617F-*negative MF. Expression of MPLW515L transforms hematopoietic cells to cytokine-independent proliferation, and results in constitutive activation of JAK-STAT signaling. The relevance of this mutation is underscored by its effect in the murine bone marrow transplant assay, as expression of MPLW515L induces myeloproliferation characterized by splenomegaly, leukocytosis, marked thrombocytosis, extramedullary hematopoeisis, and myelofibrosis. In addition, a small-molecule JAK kinase inhibitor inhibits proliferation of MPLW515L-transformed cells, suggesting that targeted inhibition of JAK-STAT signaling may be an effective therapy for both *JAK2V617F-*positive and *MPLW515L-*positive MPD.

Although the constitutively active JAK2V617F mutant kinase is present in many patients with MPD, there are a significant proportion of MF and ET patients who are *JAK2V617F-*negative. We hypothesized that activation of JAK–STAT signaling in these patients might result from activating mutations in cytokine receptors, which led to the discovery of a novel mutation in *MPL* ( *MPLW515L*) in patients with *JAK2V617F-*negative MF. The mutation is present in granulocytes but not in non-hematopoietic germline DNA, demonstrating that *MPLW515L* is a somatic mutation present in clonally derived hematopoietic cells. Moreover, the *MPLW515L* allele was not observed in a panel of normal individuals, demonstrating that it is not a common single nucleotide polymorphism. These data provide genetic evidence that *MPLW515L* is a pathogenetic mutation in this subset of *JAK2V617F-*negative MF.

In contrast to previous reports [ 13– 15], we have identified a mutant MPL receptor that results in constitutive activation of downstream signal transduction pathways. MPL signals via its association with JAK2 and TYK2, resulting in phosphorylation of both MPL and JAK2/TYK2 and consequent activation of the STAT3, STAT5, ERK/MAPK, and PI3K/AKT signaling pathways. MPLW515L activates JAK-STAT signal transduction independently of ligand binding, and, in contrast with MPLWT, confers cytokine-independent growth to 32D, Ba/F3, and UT7 cells. Moreover, Western blot analysis shows ligand-independent phosphorylation of downstream signaling proteins, which may effect both proliferation and differentiation. Finally, hematopoietic cells that express MPLW515L are hypersensitive to stimulation with TPO as assessed by cell proliferation and Western blot analysis, suggesting MPLW515L-positive hematopoietic progenitors may have a selective proliferative advantage as compared with wild-type progenitors in MF patients.

The discovery of this mutation provides a novel mechanism for activation of signal transduction in hematopoietic malignancies. Somatic mutations in MPL have not previously been described, though previous genetic and biochemical data suggest that the cytoplasmic-transmembrane junction (which includes W515) is important in its activation. These data include the germline S505N allele in *MPL* associated with familial ET [ 22, 23], the W515S mutation that has been reported as a spontaneous mutation in Ba/F3 cells stably transduced with MPL[ 24], and the observation that in vitro deletion of the RWQFP domain that includes W515 results in MPL activation [ 25]. The mechanism of activation of MPL by these alleles is not fully understood, and it will be of value to obtain structural insight into the role of the cytoplasmic transmembrane–junction region in the regulation of MPL signal transduction.

Expression of MPLW515L in murine bone marrow recapitulated many of the phenotypic characteristics of MF in humans, including atypical megakaryocytic hyperplasia, splenomegaly due to extramedullary hematopoiesis, and thrombocytosis that may be attenuated in humans by splenic sequestration [ 26, 27]. This is in contrast to murine bone marrow transplant experiments with constitutively activated tyrosine kinase alleles evaluated in our laboratory and by others, including TEL-JAK2, FLT3-ITD, and FIP1L1-PDGFRA [ 17, 28, 29], that each result in a neutrophilic MPD without involvement of the megakaryocyte lineage. It is of interest that JAK2V617F, which is associated with ET and MF in humans, results in polycythemia and leukocytosis, but not thrombocytosis, in the same murine BMT model [ 11]. Of note, absolute leukocytosis in both the JAK2V617F and the MPLW515L-mediated BMT models is higher than is typically observed in human PV and MF, respectively. Also, MPLW515L mice develop rapidly fatal disease. These differences between the murine model and the human phenotype are likely accounted for by the retroviral transduction model of disease, and is similar to what has been observed for the rapidly fatal BCR-ABL-mediated disease in the murine BMT model as compared with human CML [ 30].

MPLW515L-expressing bone marrow and spleen cells exhibit cytokine-independent colony formation, another feature of clinical ET and MF. In addition, the MPLW515L murine bone marrow transplant model shares some phenotypic characteristics with other murine models of MF, including that induced by overexpression of thrombopoietin, either in transgenic [ 31] or in bone marrow transplant models [ 32, 33], and in the transgenic GATA-1 ^low^ mouse [ 34], though the severity and chronicity of the MF phenotype varies in these different models, which, unlike the current model, are not based on somatic disease alleles observed in human MF.

MPLW515L is an attractive target for therapeutic intervention with small molecule inhibitors. Current treatments for MF include empiric therapies such as hydroxyurea or interferon, and splenectomy for symptomatic splenomegaly [ 26, 27]. Allogeneic stem cell transplantation is a viable option for some patients with MF, but most patients are diagnosed at an advanced age and are not candidates for stem cell transplantation. Recent clinical trials with thalidomide demonstrated modest efficacy in MF, largely by slowing the rate of fibrosis in these patients rather than by reversal of fibrosis [ 35]. Allogeneic BMT data does indicate that eradication of the malignant hematopoietic clone in MF does result in complete reversal of fibrosis [ 36], and suggests that drugs that target mutant alleles such as MPLW515L may be effective disease-remitting agents. JAK2 inhibitor development is well under way as a potential therapeutic strategy for treating MPD patients with the *JAK2V617F* allele. Data presented here indicate that the subset of *JAK2V617F-*negative MF patients with the *MPLW515L* mutation will also be sensitive to inhibition with small molecule JAK2 inhibitors. These findings also suggest that a more expansive genomic screen for involvement of other components of the JAK-STAT signal transduction pathway is warranted in MPD patients.

## Supporting Information

Table S1 Primer Sequences Used in This Study (25 KB DOC)

### Accession Numbers

The UniGene ( http://www.ncbi.nlm.nih.gov/entrez/query.fcgi?db=unigene) accession numbers used in this paper are *MPL* (Hs.82906 and Mm.4864), *EPOR* (Hs.127826), and *GCSFR* (Hs.524517).

## Abbreviations

EPOR: erythropoietin receptor
ET: essential thrombocytosis
FBS: fetal bovine serum
GCSFR: granulocyte-colony stimulating factor receptor
H&E: hematoxylin and eosin
MF: myelofibrosis with myeloid metaplasia
MPD: myeloproliferative disorders
MPL: thrombopoietin receptor
PV: polycythemia vera
TPO: thrombopoietin
