# Supplementary material for: MPLW515L Is a Novel Somatic Activating Mutation in Myelofibrosis with Myeloid Metaplasia
Source: PLoS Med. 2006 Jul 18;3(7):e270. doi: 10.1371/journal.pmed.0030270 (PMC1502153; doi:10.1371/journal.pmed.0030270)
Supplement: Supplementary file 1 [file pmed.0030270.st001.doc]

| Amplicon | **Forward Primer** | **Reverse Primer** |
| --- | --- | --- |
| EpoR Exon 6 | 5’-TGTAGGAGGAGCCAGGGCGAATCA | 5’-AAGAGTCTGGGTCTAGTCTGGCAT |
| EpoR Exon 7 | 5’-ATCTGTGCCTCTATGACTGGGAGT | 5’-AGCCATCATTCTGGTACAGCCACA |
| EpoR Exon 8 | 5’-TCAACAGGGCTCTGAAGCAGAAGA | 5’-GCAACCATTTGTCCAGCACCAGAT |
| Mpl Exon 1 | 5’-ggggctgtatctgacaggaa | 5’-accagcaggagagcctctta |
| Mpl Exon 2 | 5’-cgggaggggaggtagagtaa | 5’-cccacatacatccaaagcac |
| Mpl Exon 3 | 5’-atcccaggcagtgagaagaa | 5’-tatccgtgctgagctggaag |
| Mpl Exon 4 | 5’-aagctgccccaattcagc | 5’-gacgaacctaggtctggaatc |
| Mpl Exon 5 | 5’-agctgagctcctctccacag | 5’-cagtggcaagttcgagatca |
| Mpl Exon 6 | 5’-acttgccactggacaggaac | 5’-ggtttggagacaaaggaagg |
| Mpl Exon 7 | 5’-ccctggaaggaaggagttaaa | 5’-tcggtcagaagggtcagagt |
| Mpl Exon 8 | 5’-cccactgaatctgaccctgt | 5’-acatagacactttcactcctgct |
| Mpl Exon 9 | 5’-gggctctttgtgggaatctc | 5’-gcctcgcccctctcc |
| Mpl Exon 10 | 5’-TGGGCCGAAGTCTGACCCTTT | 5’-ACAGAGCGAACCAAGAATGCCTGT |
| Mpl Exon 11 | 5’-CCCTGCCCAACTTTATTGCCTTGA | 5’-GCGCTGGATTAAAGCAGATCCTGT |
| Mpl Exon 12 | 5’-TTAATCCAGCGCCTCTCCTCATCT | 5’-AGCCTAATTGTGAGGGCAGACAGA |
| GCFSR Exon 12 | 5’-CCAGCCTTCCCAACATGCATTCTA | 5’-TGCCCTTAGTGCAGAGAGAAGAAG |
| GCFSR Exon 13 | 5’-TAAGTCCCTTCCAGCCTTCCCAAA | 5’-AAGGCCTGAGTACTTGGCTTCAGA |
| GCFSR Exon 14 | 5’-TGGTACCCTTTGTGTTCCACCAGT | 5’-AAGTTGAGCAGTGGCCCAAAGACA |
| MPLW515L Genotyping | 5’-ACGTTGGATGTGACCGCTCTGCATCTAGTG | 5’-ACGTTGGATGGCGGCTGTAGTGTGCAGGA |
| Genotyping Extension Primer | 5’-CCTGCTGCTGCTGAGGT |  |

**Table S1: Primer sequences used in this study.**
